# A multicenter, retrospective observational study investigating baseline characteristics and clinical outcomes in patients with hormone-sensitive prostate cancer treated with primary androgen deprivation therapy

**DOI:** 10.1093/jjco/hyad068

**Published:** 2023-07-05

**Authors:** Satoru Taguchi, Mizuki Onozawa, Shiro Hinotsu, Taketo Kawai, Takeshi Mitomi, Satoshi Uno, Haruki Kume

**Affiliations:** Department of Urology, Graduate School of Medicine, The University of Tokyo, Tokyo, Japan; Japan Study Group of Prostate Cancer (J-CaP) Research Society, Japan; Japan Study Group of Prostate Cancer (J-CaP) Research Society, Japan; Department of Urology, International University of Health and Welfare Ichikawa Hospital, Ichikawa, Japan; Japan Study Group of Prostate Cancer (J-CaP) Research Society, Japan; Department of Biostatistics, Sapporo Medical University, Sapporo, Japan; Japan Study Group of Prostate Cancer (J-CaP) Research Society, Japan; Department of Urology, Teikyo University School of Medicine, Tokyo, Japan; Medical Oncology, Astellas Pharma Inc., Tokyo, Japan; Data Science, Astellas Pharma Inc., Tokyo, Japan; Department of Urology, Graduate School of Medicine, The University of Tokyo, Tokyo, Japan; Japan Study Group of Prostate Cancer (J-CaP) Research Society, Japan

**Keywords:** castration, degarelix, goserelin, leuprorelin, prostate cancer

## Abstract

**Objective:**

This multicenter, retrospective, observational study investigated baseline characteristics and clinical outcomes in patients with hormone-sensitive prostate cancer who received primary androgen deprivation therapy, using Japan Study Group of Prostate Cancer registry data.

**Methods:**

Among patients in the Japan Study Group of Prostate Cancer registry, those who initiated primary androgen deprivation therapy and were aged 20 years or older were enrolled in this study. The primary endpoint was time to disease progression, defined as time from primary androgen deprivation therapy initiation to either prostate-specific antigen or clinical progression. Secondary endpoints included prostate-specific antigen progression-free survival, prostate-specific antigen response (90% or greater reduction from baseline) and distribution of second-line treatment.

**Results:**

Of the 2494 patients (goserelin, *n* = 564; leuprorelin, *n* = 1148; surgical castration, *n* = 161; degarelix, *n* = 621), those who received degarelix had higher prostate-specific antigen levels and Gleason scores and were at a more advanced clinical stage than those receiving goserelin or leuprorelin. The median time to disease progression (identical to the prostate-specific antigen progression-free survival result) was not reached for goserelin and leuprorelin, 52.7 months for surgical castration and 54.0 months for degarelix. Although baseline prostate-specific antigen values in the degarelix cohort were higher than those of the leuprorelin or goserelin cohorts, prostate-specific antigen responses were not different among the three cohorts. Regarding second-line treatment, the largest patient group received degarelix followed by leuprorelin (*n* = 195).

**Conclusions:**

This study clarified patient characteristics and long-term effectiveness of primary androgen deprivation therapy in real-world clinical practice. Japanese urologists appear to select appropriate primary androgen deprivation therapy based on patient background and tumour characteristics, with degarelix largely reserved for higher risk patients.

## Teaser

This examination of real-world Japanese prescription patterns for primary androgen deprivation therapy to treat hormone-sensitive prostate cancer indicates that degarelix use is more prevalent in higher risk patients.

## Introduction

Prostate cancer is the most common cancer in Japanese males, affecting >106 000 individuals and resulting in >13 000 deaths in 2020 (1). Over the past two decades, while the incidence of prostate cancer in Japan has increased, the mortality rate has decreased (2), likely as a result of improvements in screening, diagnosis and the available treatments.

Since the publication of the landmark treatment study in 1941 (3), primary androgen deprivation therapy (PADT) has been the mainstay of therapy for metastatic hormone-sensitive prostate cancer (HSPC) worldwide. In Japan, PADT is also commonly used for patients with localized HSPC who are not eligible for definitive treatment (4), although it is currently not recommended for this purpose by Western guidelines. PADT is undertaken by either surgical castration (orchiectomy) or medical castration with a luteinizing hormone-releasing hormone (LH-RH) agonist (e.g. goserelin or leuprorelin) or antagonist (e.g. degarelix) (4). Furthermore, PADT can be combined with an antiandrogen (e.g. bicalutamide), to produce a combined androgen blockade (CAB) (5). Until recent ‘upfront’ therapies for metastatic HSPC (such as upfront PADT plus docetaxel (6)) became prevalent, CAB had been the most commonly used PADT regimen in Japan (4,7), demonstrating longer survival durations compared with LH-RH agonist monotherapy (8). The LH-RH antagonist degarelix was developed to achieve a more rapid suppression of testosterone and prostate-specific antigen (PSA) than the conventional LH-RH agonists, as well as to avoid the possibility of clinical ‘flare’ (9). Degarelix has been shown to be non-inferior to goserelin and leuprorelin in the monotherapy setting, although large comparative studies in patients receiving degarelix-based CAB are lacking (10–13).

The Japan Study Group of Prostate Cancer (J-CaP) is a research group conducting surveys of prostate cancer hormonal therapy and analyses of treatment outcomes, with the aim of developing optimal hormonal therapy guidelines for patients with prostate cancer in Japan. The J-CaP registry study began registering patients for observational analysis in January 2016, to undertake analysis of background factors at the time of diagnosis, trends in initial treatment and clinical outcomes. A prior analysis of the J-CaP study data, including patients with HSPC treated with PADT who were enrolled by the cut-off date of October 2018 (*n* = 1895), found that the majority received CAB (i.e. an LH-RH agonist or antagonist plus concomitant bicalutamide) (14). In addition, it was observed that degarelix tended to be used for the treatment of higher risk patients; degarelix-treated patients were at a more advanced clinical stage, had a higher Gleason score and a higher PSA level at diagnosis than those treated with LH-RH agonists. Moreover, the duration of degarelix treatment was shorter than that of LH-RH agonists (14).

Although the previous analysis of the J-CaP registry data provided valuable information on the contemporary treatment of prostate cancer in Japan, the follow-up period was too short to analyse long-term outcomes, including PSA progression-free survival (PSA-PFS). Therefore, a second analysis of the J-CaP registry data was planned, with a cut-off date of December 2020, to investigate patient characteristics and the long-term effectiveness of PADT in real-world clinical practice.

## Patients and methods

### Design and setting of the J-CaP registry

This was a retrospective, observational study in patients with prostate cancer who were treated with PADT using data from the J-CaP registry (UMIN000022013). The registration period of the J-CaP registry was from June 2016 to March 2019 and the participants were patients with prostate cancer in Japan who were diagnosed between January 2016 and December 2018. The registry was planned to have a follow-up period continuing until 2029. For the present analysis, the data cut-off date was 31 December 2020; on that date, the J-CaP registry encompassed 146 nationwide institutions (mostly hospitals).

Patients enrolled in the J-CaP registry had received a histopathological diagnosis of prostate cancer, and were undergoing treatment in various forms, including active surveillance, surgical treatment, radiotherapy, hormonal therapy and chemotherapy. The registry also contains information on the patient background at diagnosis, clinical staging, details of treatment, status of treatment choices and treatment outcomes to date (14). As such, it was possible to select a dataset comprised of patients who could be confirmed to have undergone PADT as their initial treatment. In the case of patients transferring to a different medical institution during follow-up, they would continue to have data entered into the registry by the original site, eliminating the possibility of duplicate records. Patients were provided with the option of opting out of registry inclusion.

### Design of this study

Patients eligible for inclusion in this study were those with HSPC, aged 20 years or older at the index date, who received PADT including surgical castration and had at least one record of treatment with androgen deprivation therapy. Patients whose age and sex data were missing or unreliable, or who initiated two or more of the treatments of interest (goserelin, leuprorelin, surgical castration and degarelix) concurrently at the index date, were excluded. Patients were categorized into four groups (goserelin, leuprorelin, surgical castration and degarelix) based on the medication/procedure used as PADT. We were unable to distinguish between treatment formulations based on the information available in the registry; thus, all degarelix-treated patients were included in a single cohort regardless of whether they might have received the 1-month or 3-month sustained release formulation.

The study was conducted in accordance with the protocol, all applicable regulations and guidelines governing study conduct, and the ethical principles that have their origin in the Declaration of Helsinki. The study was approved by the Asai Dermatology Clinic Institutional Review Board (1–14 Katabira-cho, Hodogaya-ku, Yokohama-City, Kanagawa, 240-0013, Japan) on 20 December 2021. Approvals were also sought from the Institutional Review Board or Independent Ethics Committee at each participating site, where appropriate.

### Study endpoints

The primary endpoint for this study was the time to disease progression in order to assess the long-term effectiveness of PADT. This was defined as the time from initiation of PADT to either PSA or clinical progression, according to medical record assessment by a physician. PSA progression was determined according to Prostate Cancer Clinical Trials Working Group (PCWG2) criteria (15) as an increase in PSA >25% and 2 ng/mL or more above the nadir, confirmed by progression at two time points at least 3 weeks apart. Clinical progression was determined by the treating physician based on the evidence available to them (including laboratory data, imaging and clinical symptoms). Secondary endpoints were PSA-PFS; PSA response and PSA percent change from baseline; duration of first-line treatment and distribution of second-line treatment. PSA-PFS was defined as time from initiation of PADT to PSA progression or death; PSA response was defined as ≥90% reduction in PSA from baseline, based on the data from our previous analysis which indicated that the majority of HSPC patients receiving treatment had a PSA decline of >50% (14) and duration of first-line treatment was defined as time interval from the date of initial administration of LH-RH agonist/antagonist to the date of the treatment discontinuation, loss of follow-up or death. An exploratory endpoint was to evaluate the time to disease progression according to patient baseline characteristics. Safety outcomes (adverse events) were not assessed, due to limitations relating to the information available in the J-CaP database.

### Statistical analysis

No formal sample size calculations were conducted, as no *a priori* hypotheses were tested. All eligible patients who met the inclusion and exclusion criteria were included in the overall PADT analysis set. Time-to-event analyses were conducted in patients who had initiated PADT at least 1 year prior to data cut-off and had at least one available data point after PADT initiation (effectiveness analysis set). PSA response analysis was conducted in patients who had at least one PSA measurement prior to PADT initiation and at least one PSA measurement prior to progression (PSA response analysis set).

Categorical data were reported using *n* (%), and continuous variables using mean (standard deviation [SD]) or median (interquartile range). Time-to-event analyses were conducted according to the PADT type (goserelin, leuprorelin, surgical castration or degarelix) using the Kaplan–Meier method, with the median estimated from the 50th percentile, with 95% confidence intervals (CIs). For the calculation of PSA-PFS, patients who were lost to follow-up, discontinued PADT (only for medical castration) or died without disease progression were censored at those time points. The exploratory analysis of the primary endpoint utilized a multivariate Cox proportional hazard analysis model, with patient baseline factors as covariates. For this model, adjustments were made for the treatment group (PADT) as the main effect, and several demographic factors which were considered to clinically affect the time to disease progression as potential confounding factors. These demographic factors were age, TNM classification, PSA, Gleason score at the time of diagnosis and concomitant anti-cancer drug use (CAB or non-CAB). No imputations were made for missing data. All statistical analyses were conducted using JMP Pro 15 (JMP Statistical Discovery LLC, Cary, NC, USA) and R version 4.0.0 (R Foundation for Statistical Computing, Vienna, Austria) statistical software.

## Results

### Study population

A total of 3495 HSPC patients from 146 Japanese institutions enrolled in the J-CaP registry met the study criteria for PADT and, of these, 2494 patients who were evaluable for effectiveness were included in the primary analysis (effectiveness analysis set): 564 (22.6%) received first-line treatment with goserelin, 1148 (46.0%) with leuprorelin, 161 (6.5%) underwent surgical castration and 621 (24.9%) received degarelix. Table 1 shows patient characteristics in the effectiveness analysis set according to PADT type. Overall, patients had a mean age of 76 years and a median PSA level of 23.5 ng/mL at diagnosis. Patients who received degarelix were characterized by advanced and/or high-risk disease status, including a higher PSA level (median 106.0 ng/mL) and more frequent Gleason score of 8 or more and M1 disease status than those receiving goserelin or leuprorelin. Patients who underwent surgical castration also had advanced and/or high-risk disease, albeit to a lesser extent than the degarelix cohort. The proportions of patients receiving CAB were >70% in the goserelin and leuprorelin cohorts, whereas those in the castration and degarelix cohorts were 21.7 and 56.5%, respectively.

**Table 1:** Patient characteristics according to PADT type (effectiveness analysis set, *n* = 2494)

	All PADT patients (*N* = 2494)	Goserelin (*n* = 564)	Leuprorelin (*n* = 1148)	Surgical castration (*n* = 161)	Degarelix (*n* = 621)
Age at diagnosis (years), mean (SD)	76.0 (7.3)	75.9 (6.8)	76.8 (6.6)	78.2 (7.3)	73.9 (8.3)
PSA at diagnosis (ng/mL), median (IQR)	23.5 (9.8–127.1)	16.4 (8.4–52.0)	16.3 (8.5–51.3)	91.7 (15.5–355.0)	106.0 (22.4–522.2)
Gleason score, *n* (%)					
2–6	151 (6.1)	39 (6.9)	93 (8.1)	11 (6.8)	8 (1.3)
7	694 (27.8)	198 (35.1)	387 (33.7)	28 (17.4)	81 (13.0)
8–10	1644 (65.9)	327 (58.0)	665 (57.9)	121 (75.2)	531 (85.5)
Missing	5 (0.2)	0	3 (0.3)	1 (0.6)	1 (0.2)
T stage, *n* (%)					
T0	2 (0.1)	1 (0.2)	0	1 (0.6)	0
T1	353 (14.2)	101 (17.9)	200 (17.4)	12 (7.5)	40 (6.4)
T2	909 (36.4)	240 (42.6)	482 (42.0)	46 (28.6)	141 (22.7)
T3	870 (34.9)	168 (29.8)	358 (31.2)	71 (44.1)	273 (44.0)
T4	335 (13.4)	49 (8.7)	99 (8.6)	29 (18.0)	158 (25.4)
Missing	25 (1.0)	5 (0.9)	9 (0.8)	2 (1.2)	9 (1.4)
N stage, *n* (%)					
N0	1769 (70.9)	458 (81.2)	918 (80.0)	103 (64.0)	290 (46.7)
N1	687 (27.5)	96 (17.0)	215 (18.7)	53 (32.9)	323 (52.0)
Nx	35 (1.4)	10 (1.8)	13 (1.1)	5 (3.1)	7 (1.1)
Missing	3 (0.1)	0	2 (0.2)	0	1 (0.2)
M stage, *n* (%)					
M0	1665 (66.8)	442 (78.4)	902 (78.6)	88 (54.7)	233 (37.5)
M1	825 (33.1)	120 (21.3)	244 (21.3)	73 (45.3)	388 (62.5)
Missing	4 (0.2)	2 (0.4)	2 (0.2)	0	0
Clinical stage, *n* (%)					
I	693 (27.8)	205 (36.3)	408 (35.5)	28 (17.4)	52 (8.4)
II	351 (14.1)	101 (17.9)	198 (17.2)	12 (7.5)	40 (6.4)
III	366 (14.7)	81 (14.4)	194 (16.9)	28 (17.4)	63 (10.1)
IV	1053 (42.2)	169 (30.0)	331 (28.8)	91 (56.5)	462 (74.4)
Missing	31 (1.2)	8 (1.4)	17 (1.5)	2 (1.2)	4 (0.6)
J-CAPRA score, *n* (%)					
0–2	884 (35.4)	254 (45.0)	538 (46.9)	33 (20.5)	59 (9.5)
3–7	889 (35.6)	208 (36.9)	402 (35.0)	57 (35.4)	222 (35.7)
8–12	657 (26.3)	88 (15.6)	183 (15.9)	63 (39.1)	323 (52.0)
Missing	64 (2.6)	14 (2.5)	25 (2.2)	8 (5.0)	17 (2.7)
ADT type, *n* (%)					
CAB	1616 (64.8)	413 (73.2)	817 (71.2)	35 (21.7)	351 (56.5)
Non-CAB	595 (23.9)	101 (17.9)	233 (20.3)	67 (41.6)	194 (31.2)
Missing	283 (11.3)	50 (8.9)	98 (8.5)	59 (36.6)	76 (12.2)
Concomitant anti-cancer drug, *n* (%)					
Yes	1616	413	817	35	351
Abiraterone	11 (0.7)	1 (0.2)	1 (0.1)	0	9 (2.6)
Bicalutamide	1595 (98.7)	411 (99.5)	812 (99.4)	35 (100.0)	337 (96.0)
Chlormadinone	6 (0.4)	1 (0.2)	2 (0.2)	0	3 (0.9)
Flutamide	4 (0.2)	0	2 (0.2)	0	2 (0.6)

ADT, androgen deprivation therapy; CAB, combined antiandrogen blockade; J-CAPRA, Japan Cancer of the Prostate Risk Assessment; IQR, interquartile range; PADT, primary androgen deprivation therapy; PSA, prostate-specific antigen; SD, standard deviation.

### Time to disease progression (primary endpoint)

**Figure 1:**
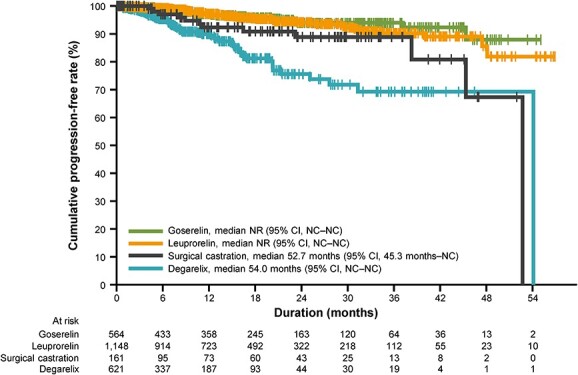
Time to disease progression (primary endpoint) according to the type of primary androgen deprivation therapy. The vertical whiskers indicate censoring. CI, confidence interval; NC, not calculable; NR, not reached.

The median time to disease progression (effectiveness analysis set, *n* = 2494) was not reached for goserelin or leuprorelin, was 52.7 months for surgical castration and was 54.0 months for degarelix (Fig. 1). The cumulative disease progression-free rates at 2 years were 94.0% (95% CI, 90.7–96.2) with goserelin, 94.2% (95% CI, 92.1–95.8) with leuprorelin, 88.9% (95% CI, 79.6–94.3) with surgical castration and 75.5% (95% CI, 68.2–81.9) with degarelix (Table 2).

**Table 2:** Time to disease progression for all patients (effectiveness analysis set, *n* = 2494) and according to the M stage (subgroup analysis)

	Overall PADT	Goserelin	Leuprorelin	Surgical castration	Degarelix
All patients, *N*	2494	564	1148	161	621
Event, *n* (%)	147 (5.9)	22 (3.9)	53 (4.6)	12 (7.5)	60 (9.7)
PSA progression only, *n* (%)	96 (3.8)	15 (2.7)	39 (3.4)	5 (3.1)	37 (6.0)
Clinical progression only, *n* (%)	12 (0.5)	1 (0.2)	3 (0.3)	2 (1.2)	6 (1.0)
PSA progression and clinical progression, *n* (%)	39 (1.6)	6 (1.1)	11 (1.0)	5 (3.1)	17 (2.7)
Time to progression (months), median (95% CI)	NR (NC–NC)	NR (NC–NC)	NR (NC–NC)	52.7 (45.3–NC)	54.0 (NC–NC)
1-year progression-free rate, % (95% CI)	95.5 (94.4–96.4)	97.3 (95.2–98.4)	97.2 (95.8–98.1)	92.4 (84.8–96.3)	90.1 (86.4–92.8)
2-year progression-free rate, % (95% CI)	91.0 (89.2–92.5)	94.0 (90.7–96.2)	94.2 (92.1–95.8)	88.9 (79.6–94.3)	75.7 (68.2–81.9)
Stage M0, *n*	1665	442	902	88	233
Event, *n* (%)	52 (3.1)	12 (2.7)	27 (3.0)	4 (4.5)	9 (3.9)
PSA progression only, *n* (%)	40 (2.4)	8 (1.8)	22 (2.4)	2 (2.3)	8 (3.4)
Clinical progression only, *n* (%)	4 (0.2)	0	2 (0.2)	1 (1.1)	1 (0.4)
PSA progression and clinical progression, *n* (%)	8 (0.5)	4 (0.9)	3 (0.3)	1 (1.1)	0 (0.0)
Time to progression (months), median (95% CI)	NR (NC–NC)	NR (NC–NC)	NR (NC–NC)	52.7 (45.3–NC)	NR (NC–NC)
1-year progression-free rate, % (95% CI)	98.3 (97.4–98.9)	98.0 (95.9–99.1)	98.5 (97.2–99.2)	98.4 (89.4–99.8)	97.7 (93.0–99.3)
2-year progression-free rate, % (95% CI)	96.3 (94.9–97.4)	96.4 (93.1–98.1)	96.8 (94.8–98.0)	96.3 (86.4–99.1)	92.6 (83.6–96.9)
Stage M1, *n*	825	120	244	73	388
Event, *n* (%)	95 (11.5)	10 (8.3)	26 (10.7)	8 (11.0)	51 (13.1)
PSA progression only, *n* (%)	56 (6.8)	7 (5.8)	17 (7.0)	3 (4.1)	29 (7.5)
Clinical progression only, *n* (%)	8 (1.0)	1 (0.8)	1 (0.4)	1 (1.4)	5 (1.3)
PSA progression and clinical progression, *n* (%)	31 (3.8)	2 (1.7)	8 (3.3)	4 (5.5)	17 (4.4)
Time to progression (months), median (95% CI)	54.0 (48.1–NC)	NR (NC–NC)	NR (48.1–NC)	38.3 (23.2–NC)	54.0 (NC–NC)
1-year progression-free rate, % (95% CI)	88.5 (85.3–91.0)	94.1 (86.4–97.5)	91.5 (86.1–95.0)	80.6 (62.5–91.1)	85.2 (79.8–89.4)
2-year progression-free rate, % (95% CI)	75.3 (69.9–80.1)	82.3 (68.5–90.9)	82.2 (74.0–88.3)	70.5 (45.0–87.5)	65.8 (55.6–74.7)

CI, confidence interval; NC, not calculable; NR, not reached.

In a subgroup analysis of the primary endpoint, time to progression according to the M stage was assessed (Table 2). In patients with M0 disease (the majority of patients in the goserelin, leuprorelin and surgical castration cohorts, and the minority in the degarelix cohort), the cumulative progression-free rates at 2 years were all >90%. In contrast, the cumulative progression-free rates at 2 years in patients with M1 disease were >80% in the goserelin and leuprorelin cohorts, whereas those in the castration and degarelix cohorts were 70.5 and 65.8%, respectively.

In the exploratory multivariate Cox proportional hazard analysis of factors influencing time to disease progression, greater age (75 years or older), higher Gleason score (8 or greater) and advanced clinical stage (T3–4, N1, M1) were all associated with shorter time to progression (Table 3). Treatment with goserelin or leuprorelin was associated with longer time to progression than degarelix, even after adjustment for potential confounding factors indicating unfavourable disease status at diagnosis.

**Table 3:** Multivariate Cox proportional hazards analysis of factors influencing time to disease progression (effectiveness analysis set, *n* = 2494)

Demographic factors	*n*	HR (95% CI)
Treatment group		
Goserelin	500	0.5 (0.3–0.9)
Leuprorelin	1029	0.6 (0.4–0.9)
Surgical castration	96	0.8 (0.4–1.5)
Degarelix	530	Reference
Age at diagnosis (years)		
<75	837	Reference
≥75	1318	1.5 (1.0–2.1)
PSA at diagnosis (ng/mL)		
<20	1021	Reference
≥20 to <100	560	1.4 (0.8–2.5)
≥100	574	1.4 (0.8–2.6)
Gleason score		
≤7	754	Reference
≥8	1401	1.9 (1.1–3.5)
T stage		
T1–2	1121	Reference
T3–4	1034	1.6 (1.0–2.6)
N stage		
N0	1570	Reference
N1	585	2.6 (1.7–3.9)
M stage		
M0	1481	Reference
M1	674	2.2 (1.4–3.4)
ADT type		
CAB	1580	Reference
Non-CAB	575	1.3 (0.9–2.0)

HR, hazard ratio.

The model was adjusted for the treatment group (PADT) as the main effect, and several demographic factors (age, TNM classification, PSA, Gleason score at the time of diagnosis and concomitant anti-cancer drug use [CAB or non-CAB]) as potential confounding factors.

### Secondary endpoints

**Figure 2:**
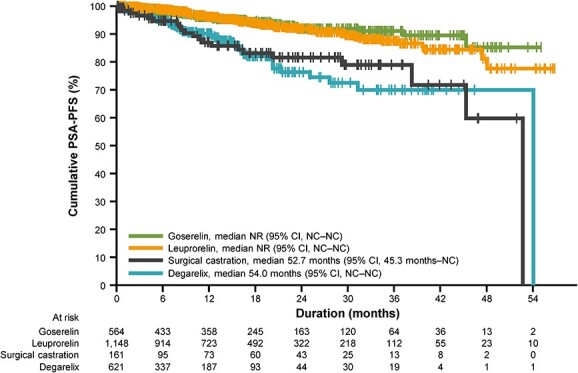
Kaplan–Meier analysis of PSA-PFS according to the type of primary androgen deprivation therapy. The vertical whiskers indicate censoring. PFS, progression-free survival; PSA, prostate-specific antigen.

Median PSA-PFS (effectiveness analysis set) was not reached for goserelin and leuprorelin, was 52.7 months for surgical castration and was 54.0 months for degarelix (Fig. 2). The cumulative PSA-PFS rate at 2 years was 92.0% (95% CI, 88.4–94.5) with goserelin, 92.0% (95% CI, 89.6–93.8) with leuprorelin, 81.6% (95% CI, 72.2–88.4) with surgical castration and 76.4% (95% CI, 69.0–82.5) with degarelix (Table 4).

**Table 4:** Summary of secondary endpoints

	Overall PADT	Goserelin	Leuprorelin	Surgical castration	Degarelix
PFS^a^, *n*	2494	564	1148	161	621
Event, *n* (%)	184 (7.4)	31 (5.5)	73 (6.4)	21 (13.0)	59 (9.5)
PSA progression only, *n* (%)	96 (3.8)	15 (2.7)	39 (3.4)	5 (3.1)	37 (6.0)
PSA progression and clinical progression, *n* (%)	39 (1.6)	6 (1.1)	11 (1.0)	5 (3.1)	17 (2.7)
Death, *n* (%)	49 (2.0)	10 (1.8)	23 (2.0)	11 (6.8)	5 (0.8)
Duration of PFS (months), median (95% CI)	NR (54.0–NC)	NR (NC–NC)	NR (NC–NC)	52.7 (45.3–NC)	54.0 (NC–NC)
1-year PSA-PFS rate, % (95% CI)	94.5 (93.3–95.4)	96.2 (94.0–97.6)	96.2 (94.7–97.3)	87.0 (78.8–92.4)	90.3 (86.7–92.9)
2-year PSA-PFS rate, % (95% CI)	89.0 (87.1–90.6)	92.0 (88.4–94.5)	92.0 (89.6–93.8)	81.6 (72.2–88.4)	76.4 (69.0–82.5)
PSA response^a^, *n*	1684	398	842	100	344
Yes, *n* (%)	1528 (90.7)	377 (94.7)	781 (92.8)	83 (83.0)	287 (83.4)
No, *n* (%)	156 (9.3)	21 (5.3)	61 (7.2)	17 (17.0)	57 (16.6)
Percent reduction in PSA from baseline at maximum reduction during PADT, mean (SD)	95.1 (47.7)	96.7 (29.3)	97.3 (10.7)	77.4 (179.1)	93.0 (22.5)
Duration of treatment^a^, *n*	3300	868	1595	-	837
Event, *n* (%)	594 (18.0)	106 (12.2)	146 (9.2)	-	342 (40.9)
Death, *n* (%)	95 (2.9)	18 (2.1)	52 (3.3)	-	25 (3.0)
Withdrawal, *n* (%)	499 (15.1)	88 (10.1)	94 (5.9)	-	317 (37.9)
Duration of treatment (months), median (95% CI)	NR (NC–NC)	NR (NC–NC)	NR (NC–NC)	-	17.4 (15.0–24.1)
1-year continuation rate, % (95% CI)	84.2 (82.7–85.6)	90.7 (88.3–92.7)	93.2 (91.6–94.5)	-	60.0 (56.1–63.8)
2-year continuation rate, % (95% CI)	76.8 (74.9–78.6)	84.7 (81.4–87.6)	88.7 (86.5–90.6)	-	45.6 (41.3–50.1)

^a^PFS was evaluated in the effectiveness analysis set; PSA response was evaluated in the PSA response analysis set; duration of treatment was evaluated in the overall PADT analysis set excluding patients who underwent surgical castration.

PSA response rates (PSA response analysis set, *n* = 1684) were 377/398 (94.7%) for goserelin, 781/842 (92.8%) for leuprorelin, 83/100 (83.0%) for surgical castration and 287/344 (83.4%) for degarelix. The mean (SD) percent reductions from baseline in PSA level at maximum reduction during PADT were 96.7% (29.3) with goserelin, 97.3% (10.7) with leuprorelin, 77.4% (179.1) with surgical castration and 93.0% (22.5) with degarelix (Table 4). Although baseline PSA values were higher in the degarelix cohort than in the leuprorelin and goserelin cohorts, the PSA responses during PADT were not different among any of the three LH-RH agonist/antagonist treatments.

**Figure 3:**
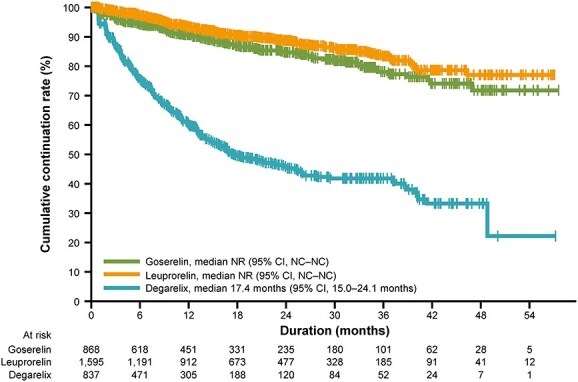
Kaplan–Meier analysis of duration of treatment according to the first-line primary androgen deprivation therapy (*n* = 3300). The vertical whiskers indicate censoring.

The median duration of treatment was evaluated in the overall population of 3495 HSPC patients who received PADT; 195 patients who underwent surgical castration were excluded, leaving 3300 patients for inclusion in this analysis. The median duration of treatment was not reached for goserelin or leuprorelin, and was 17.4 months for degarelix (Fig. 3, Table 4).

A total of 520 patients received second-line treatment, which comprised degarelix in 27 (5.2%) patients, goserelin in 179 (34.4%), leuprorelin in 282 (54.2%) and surgical castration in 32 (6.2%). A breakdown of second-line treatment according to the first-line PADT agent is shown in Table 5. The largest patient group comprised those who received degarelix as first-line PADT and subsequently switched to leuprorelin as the second-line treatment (*n* = 195).

**Table 5:** Summary of second-line treatment according to the first-line PADT agent

First-line PADT agent	*n*	Median duration, months	Second-line treatment	*n* (%)
Goserelin	91	8.3	Goserelin	6 (6.6)
			Leuprorelin	71 (78.0)
			Surgical castration	8 (8.8)
			Degarelix	6 (6.6)
Leuprorelin	96	9.1	Goserelin	51 (53.1)
			Leuprorelin	16 (16.7)
			Surgical castration	12 (12.5)
			Degarelix	17 (17.7)
Degarelix	333	5.5	Goserelin	122 (36.6)
			Leuprorelin	195 (58.6)
			Surgical castration	12 (3.6)
			Degarelix	4 (1.2)

## Discussion

In this study using patient data collected in the J-CaP registry, we investigated patient characteristics and long-term effectiveness of PADT in real-world clinical practice. Notably, we found that patients treated with degarelix tended to have unfavourable disease characteristics at the time of diagnosis, including higher PSA level, higher Gleason score and more advanced clinical stage compared with other treatment cohorts. As a result, patients in the degarelix cohort had a shorter time to progression and lower 2-year progression-free rate than those who received goserelin or leuprorelin. These results are in line with a previous analysis of patients in the J-CaP registry, in which degarelix-treated patients had the highest median PSA (116.7 ng/mL) and the highest proportions of stage IV disease (72.9%) and Gleason score 9–10 (59.7%) compared with the other PADT types (14).

The results of our analysis found that the 1-year progression-free rate in the degarelix cohort was 90.1%. In a prospective phase 3 study of degarelix and goserelin conducted in China, 1-year progression-free rates were evaluated as a secondary endpoint, and were reported to be 81.5% in the degarelix cohort and 71.7% in the goserelin cohort (12). While the disease characteristics of the degarelix-treated patients were similar between our study and the Chinese study (baseline PSA was 106.0 and 89.3 ng/ml, and the proportion with M1 disease was 62.5 and 63%, respectively), the 1-year progression-free rate in our study was noticeably higher. A direct comparison between the two studies is not possible, due to differences in study design, the event definitions used, patient demographics and the method of follow-up. Nonetheless, we can speculate that the higher proportion of CAB use in this study (comprising approximately two-thirds of the patients evaluated) may be partially responsible for the difference in 1-year progression-free rates between these studies.

Although the 2-year progression-free rate of the degarelix cohort (75.7%) in this study was lower than the other treatment cohorts (88.9% or greater), this could have been attributable to the unfavourable disease characteristics in patients chosen to receive degarelix. The subgroup analysis according to the M stage demonstrated that outcomes in patients with M0 disease were similar across treatment groups, with 2-year progression-free rates of 90% or more. However, given that the majority of degarelix-treated patients had high-risk M1 disease, this imbalance may have given rise to an outcome bias favouring LH-RH agonists (goserelin or leuprorelin) in the overall population. Other factors associated with shorter time to disease progression included greater age, higher Gleason score and more advanced disease stage. Notably, the results of the exploratory multivariate Cox regression analysis found an association between a shorter time to disease progression and degarelix treatment, even after adjustment for unfavourable disease status at diagnosis. However, it remains unclear at this time whether degarelix increased the risk of progression or whether the difference in time to progression between the LH-RH agonists and degarelix was due to the presence of residual risk factors that were not identified in this analysis.

Although baseline PSA values in the degarelix cohort were higher than those in the cohorts receiving goserelin or leuprorelin, we did not observe any notable differences in PSA response during PADT among these treatment groups. This is in contrast to our previous analysis of the J-CaP study data, in which the PSA response rate of the leuprorelin group was higher than the other treatment groups (14). This discrepancy is likely due to the greater number of patients in each treatment cohort, the longer follow-up duration and the slightly different definition of PSA response (≥90% reduction from baseline) used in this study.

Despite the longer follow-up period compared with the prior analysis, the median duration of treatment was still not reached for goserelin and leuprorelin, and was 17.4 months for degarelix. One potential reason for the shorter duration of treatment for degarelix may be that patients switched to other treatments (e.g. LH-RH agonists) due to adverse events; however, we cannot confirm this supposition as safety events were not collected in this registry. Overall, 1- and 2-year continuation rates (84.2 and 76.8%, respectively) were similar to those of our previous report (83.7 and 75.5%, respectively) (14), with lower rates in the degarelix cohort than in the goserelin and leuprorelin cohorts. In the analysis of second-line treatment, the largest group of patients were those who switched from first-line degarelix to second-line leuprorelin; this result was in line with our prior analysis (14).

Limitations of this analysis include that the data may not be generalizable to primary care or smaller clinics, because the information was collected from relatively large hospitals. In addition, the data in the J-CaP registry were entered by physicians according to the content of the available medical records, and some information may be missing. For example, testosterone levels were not known for most patients, imaging data were not routinely available for all patients, detailed disease progression could not be assessed and the PSA response could have been misidentified as a result of infrequent measurement. We were also unable to distinguish between different product formulations (e.g. 1-month or 3-month sustained release) based on the registry data available. Details of demographic and disease characteristics were not exhaustive, and omissions may have restricted our ability to identify additional confounding risk factors affecting treatment outcomes. The reasons for withdrawal of LH-RH products and administration records (including changes in dose) were not collected in this study and could not, therefore, be evaluated. Finally, some patients who were transferred to other medical institutions during treatment may have been lost to follow-up. However, by including ~10% of all Japanese patients with prostate cancer in the J-CaP registry, this study provides an important and much-needed insight into long-term effectiveness of various PADT modalities for prostate cancer in Japan.

In conclusion, using data from the J-CaP registry study to examine the patient characteristics and long-term effectiveness of PADT in real-world clinical practice, this study indicated that urologists in Japan currently make their clinical decisions to select appropriate PADT based on patient background and tumour characteristics, with degarelix largely reserved for higher risk patients. Degarelix-treated patients were found to have a shorter time to progression and lower 2-year progression-free rate compared with goserelin- or leuprorelin-treated patients. Given that outcomes in lower risk patients with non-metastatic disease were similar between degarelix and the LH-RH agonists (goserelin or leuprorelin), and there were no differences in PSA response between these agents, the seemingly inferior outcomes of degarelix in this study are unlikely to be due to its antitumor efficacy, but more likely reflect the real-world prescription pattern of PADT for HSPC.

## Data Availability

The data underlying this article were provided by the Japan Study Group of Prostate Cancer (J-CaP). Inquiries and requests for data sharing should be made at https://j-cap.jp/.
